# Challenges in identifying large germline structural variants for clinical use by long read sequencing

**DOI:** 10.1016/j.csbj.2019.11.008

**Published:** 2019-12-23

**Authors:** Barbara Jenko Bizjan, Theodora Katsila, Tine Tesovnik, Robert Šket, Maruša Debeljak, Minos Timotheos Matsoukas, Jernej Kovač

**Affiliations:** a Clinical Institute of Special Laboratory Diagnostics, University Children’s Hospital, UMC, Ljubljana, Slovenia; b Institute of Chemical Biology, National Hellenic Research Centre, Athens, Greece; c Cloudpharm P.C., Greece

**Keywords:** Structural variations, Human genetics, Long reads sequencing, Theranostics

## Abstract

Genomic structural variations, previously considered rare events, are widely recognized as a major source of inter-individual variability and hence, a major hurdle in optimum patient stratification and disease management. Herein, we focus on large complex germline structural variations and present challenges towards target treatment via the synergy of state-of-the-art approaches and information technology tools. A complex structural variation detection remains challenging, as there is no gold standard for identifying such genomic variations with long reads, especially when the chromosomal rearrangement in question is a few Mb in length. A clinical case with a large complex chromosomal rearrangement serves as a paradigm. We feel that functional validation and data interpretation are of outmost importance for information growth to be translated into knowledge growth and hence, new working practices are highlighted.

## 1 Introduction

Human genome carries a median of 18.4 Mb of large structural variations (SVs) (>50 kb) per diploid genome. Multi-allelic copy number variations (CNV) and duplications (median length larger than 10 kb) are prominent [1]. To date, despite technological advances and a rich repertoire of sequencing methods, the characterization of large complex structural variation with exact breakpoints remains costly and of note, highly demanding.

In the clinic, such hurdles need to be overcome. Indeed, quality of diagnosis for rare complex structural rearrangements would be remarkably improved, if exact breakpoints could be detected with base-pair-resolution. Further, accurate breakpoint mapping, gene identification with high accuracy, precision, and robustness for those being rearranged may empower clinical diagnosis. A clear insight into the pathogenesis of the genomic landscape sheds light into the molecular mechanisms of the genetic rearrangement in question.

A clinical phenotype of severe developmental delay (DD), possibly indicating a nested or large SV, may serve as a paradigm. For someone to explore the molecular mechanisms that generated such a SV, a multi-step approach is presented that consists of cytogenetic pre-screening, next generation sequencing (NGS) of a region of interest, followed by clinical phenotype interpretation and conformational SV analysis. Cytogenetic approaches (or optical mapping) allow for low resolution genome screening. Notwithstanding, insertions and deletions can also be detected by CNV analysis (short-read sequencing). Next, NGS enables the in-depth characterization for the genome regions of interest. Due to a high number of false positive variant calls, emphasis may be put on the SVs that are validated by cytogenetics. Using NGS, breakpoints can be detected along with the genes or their parts involved in the rearrangement of interest. The latter may be validated further by Sanger sequencing and/or long-range PCR coupled by NGS. Functional studies, although at their infancy, may validate datasets and hypotheses and enable clinical insights.

Herein, we build on the principles and strategies of clinical cytogenetics and present encountered challenges in the identification of large germline structural variants. Long read sequencing technologies hold promise as a theranostics roadmap and for this, a specific technical aspect of a clinical case with known complex structural rearrangement was selected for the demonstration. State-of-the-art methodologies were employed and integrated to allow for high diagnostic accuracy. To this end, the added value of multi-omics and 3D cell co-cultures is a potenital path towards better-informed decision-making in the clinic and clinically relevant biomarkers.

## 2 Cytogenetic approaches for exploring disease phenotypes

In the last 62 years, since the identification of the exact chromosome number in a diploid human cell by Tjio and Levan in 1956 [2], great advances occurred in the field of cytogenetics, not only in terms of the technology itself, but also highlighting genotype-to-phenotype associations via the study of chromosomal structural variations. A great plethora of different staining and banding techniques emerged, together with the development of the fluorescent *in situ* hybridisation (FISH) and comparative genomic hybridisation (CGH) methods to interrogate the structural phenomena of the human genome [3].

Chromosome G-banding, historically, has been the most widely adopted chromosome banding and staining technique, based on the partial trypsin digestion of the chromosomal protein scaffold followed by Giemsa staining of fixed metaphases [4]. The characteristic bright and dark chromosome bands were associated with chromatin types; bright bands represented lightly packed and usually actively transcribing euchromatin, whereas heterochromatin (densely packed, mostly inactive) was observed by dark bands. The signature sequence of those bright and dark bands was dependent on the level of chromosome condensation and thus, it was directly associated with the resolution of the analysis (smaller, more densely packed chromosomes yielded less bands of low resolution, when compared to the longer, less condensed chromosomes). Overall, the resolution of the analysis was highly dependent on the chromosome region *per se*, except for the aforementioned methodological aspects.

In 1985, Landegent and colleagues mapped the first single-copy human gene to a specific genomic location using FISH [5]. The latter, soon, became one of the gold-standard methods to explore chromosomal loci of interest as well as smaller, hard-to-observe structural variants by banding techniques. Deletion and duplication syndromes, such as DiGeorge or Prader-Willi and Beckwith-Wiedemann or Potocki-Lupski syndromes, respectively as well as other microdeletion/duplication events affecting human health were routinely diagnosed using FISH, being a widely established method in the field of clinical genetics [6].

In brief, when performing a FISH experiment, multiple specific chromophore labeled oligonucleotide probes, complementary to the region of interest (ROI), are applied to the fixed metaphase slides. During the hybridisation process, which involves partial DNA denaturation and renaturation, the probes attach to their specific location along the ROI. After the removal of excessive non-bound and poorly bound probes and addition of a counter-stain to visualise chromosomes and/or nuclei, the ROI are usually visualized as coloured dots on the counter-stained chromosomes or interphase nuclei by a fluorescent microscope system. Upon analysis, depending on the number of ROI copies present in the chromosomes studied, there may be single, double or multiple signals detected in the metaphase (or interphase) nuclei. Overall, FISH is a relatively straightforward method, when interrogating relatively simple structural rearrangements using up to three different probes. Challenges arise when mapping complex chromosomal rearrangements utilising multiple probes is desired, accompanied by technical and financial burdens, as expensive equipment (additional optical filters) and technical skills become indispensable. It should be also noted that there may be a profound crosstalk among multiple probes, as their emission spectra may be too close to each other and hence, available filters cannot eliminate such non-specific signals [7]. Consequently, the number of the available fluorescent filters of the microscope system limits the maximum number of the probes applied per FISH experiment. Adding further to the complexity and cost of FISH experiments, if prior knowledge is not present regarding the SV of interest, mutli-colour FISH (mFISH), spectral karyotyping FISH (SKY FISH) and multi-colour banding FISH (mBAND FISH) approaches are required [8].

For larger chromosomal CNV (deletions and insertions) screening, CGH (comparative genomic hybridization), which is also known as metaphase CGH, was the first method employed. CGH is based on the comparison of a fluorescence-labeled control vs. the metaphase chromosomes of a sample, hybridized on glass slides and analyzed by fluorescence microscopy [9]. The method has several limitations due to cell culture demands and non-specific fluorescent signals during imaging, while it is labor-intensive and hard to standardize due to its relatively low resolution. For this, BAC-based array CGH has been developed, printing chromosomal regions on a glass slide. However, the oligo-based array CGH (aCHG) has been the method that revolutionized molecular cytogenetics. When performing such experiment, DNA samples are labeled with fluorescence dyes hybridized on a matrix of synthetic short oligo-nucleotides, which are synthesized *in-situ* on a glass slide [10]. Data analysis was supported further by automation, even during capturing the microscopic images of interest [11]. Today, there are various resolution arrays on the market, suitable for several types of analysis, with the highest resolution of 200 b obtained in SNP arrays. Nevertheless, CGH cannot be employed for the detection of inversions, balanced translocations, reciprocal insertions or mosaicism, while this method cannot locate the SV regions, which are not mapped by the array probes used [12].

## 3 Long read sequencing revolutionizes medical genetics

Oxford Nanopore Technologies (ONT) has introduced nanopore DNA sequencing [13], while Pacific Bioscences (PacBio) commercialized long-read single-molecule sequencing using single-molecule real time (SMRT) technique [14]. These long-read sequencing technologies can produce reads of approximately 10 kb in length, with many being of over 100 kb in length, while the maximum read length may be over 1 Mb [15].

Long read sequencing has the potential to capture clinically important large genomic structural rearrangements as well as repetitive sequences and single nucleotide variants, overcoming the limitations of NGS short reads, which produce reads spanning 50–600 bp, as the detection of SVs from short read data often suffers from low sensitivity (30–70%) and high false discovery rate (up to 85%) [15]. On the other hand, and despite recent improvements in computational tools and ONT chemistry, which result in higher data yields, long read sequencing exhibits a high error rate, in the range of 5%–15% on a single nucleotide resolution [16], [17]. PacBio technology produces data of better quality, overall, although with a 13–15% error rate [18]. Yet, new releases of bioinformatics tools, almost on a monthly basis, lead to single nucleotide variant calling and SV breakpoints identification of improved quality and precision.

Today, two main computational approaches prevail: reference-based alignment of reads with structural variation calling and *de novo assembly* followed by reference-based assembly alignment (Table 1). The former is advantageous in terms of lower coverage requirements (∼15X) towards the identification of heterozygous variants, whereas the latter resolves the full spectrum of human genome variation, including large SVs [15].

**Table 1:** Bioinformatics methods for the discovery and identification of structural variants by long read sequencing.

Bioinformatics analysis	Selected methods	References	Sequencing technology
Reference based alignment of reads with structural variation calling			
Reads alignment	NGMLR	Sedlazeck, Rescheneder, et al. [21]	ONT or PacBio
	Minimap2	Li [20]	
Variant calling	Sniffles	Sedlazeck, Rescheneder, et al. [21]	
	SVIM	Heller and Vingron [22]	
	SMRT-SV	Huddleston et al. [24]	PacBio
	PBHoney	English et al. [23]	
Visualization	IGV	Robinson et al. [25]	ONT or PacBio
	Ribbon	Nattestad, Chin, et al. [26]	
*De novo assembly* followed by reference-based assembly alignment			
Assembly	Canu	Koren et al. [30], [31]	ONT or PacBio
	Wtdbg2	Ruan and Li [27]	
	FALCON	Chin et al. [29]	PacBio
Assembly alignment and visualization	MUMer	Marcais et al. [32]	ONT or PacBio
	QUAST	Gurevich et al. [33]	
Assembly-based SV detection	Assemblitics	Nattestad and Schatz [34]	

### 3.1 Reference-based alignment of reads with structural variation calling

Currently, the highest accuracy in SV calling has been achieved by the CoNvex Gap-cost align Ments for Long Reads (NGMLR) mapper or the Minimap2 aligner, followed by Sniffles or SVIM variant callers [19], [20]. As shown in Table 1 these information technology tools can be used for both ONT and PacBio reads.

NGMLR was designed to quickly and correctly align the reads of interest, including those spanning (complex) SVs. NGMLR uses the convex gap-cost scoring model to accurately align long reads across small indels that commonly occur as sequencing errors. Moreover, larger and complex SVs are captured through spot-read alignments [21]. Minimap2 aligner is faster than NGMLR as it works like most whole genome aligners (seed-chain-align procedure). In short, Minimap2 indexes the minimizers of the reference and stores a list of locations of the minimizer copies as a value. Then, Minimap2 takes query minimizers and finds exact matches to the reference for each query sequence. A set of collinear matches to the reference are identified as chains. Minimap2, next, performs a dynamic programming-based global alignment between adjacent matches to the reference in a chain [20]. Sniffles is a variant caller that detects all types of SVs from long read alignments: indels, duplications, inversions, translocations, and nested events. It was made as a complementary tool to the NGMLR aligner, but it can be used with any aligner. For the detection of large and complex events, Sniffles uses split-read information, while small indels that can be spanned within a single read are detected by within-alignment scanning. Additionally, Sniffles can reconstruct the haplotype structure of a sample by read-based phasing of SVs and thus determines adjacent or nested events [21]. Another variant caller that can be used for large nested structural variants is Structural Variant Identification Method (SVIM). SVIM can detect deletions, insertions, tandem and interspersed duplications, inversions and novel element insertions. It consists of three components: collection, clustering and combination of structural variant signatures from read alignments [22].

Within PacBio reads, large SVs can be identified by PBHoney or SMRT-SV, too. PBHoney comprises two variant identifications approaches; a) PBHoney-Spots considers intra-read discordance by a subsequent increase or decrease in error along the reference sequence and b) PBHoney-Tails identifies structural variants by realigning soft-clipped tails of long reads (>10,000 bp) to the reference genome [23]. SMART-SV identifies signatures of putative structural variation from the alignments of raw reads to the reference genome, and then, it generates local assemblies from regions with structural variation signatures [24].

Even though read aligners as Minimap2 and NGMLR explicitly take large SVs > 50 bp into account, it is still not clear how these aligners and variant callers are detecting complex variants spanning few Mbp lengths with multiple structural rearrangement events. SV detection and identification is challenging using current analytical approaches, especially when SVs are longer than the average read length. For SVs that were identified using Sniffles after NGLMR alignment, the SV validation status (per length of SVs) failed to detect any true variants spanning over 7.5 kb [19]. To identify heterozygous SVs remains also challenging. SMRT-SV analysis of SVs on a pseudodiploid genome, which was constructed *in silico* by merging two haploids, have missed more than a half (∼59%) of the heterozygous SVs [24].

**Fig. 1:**
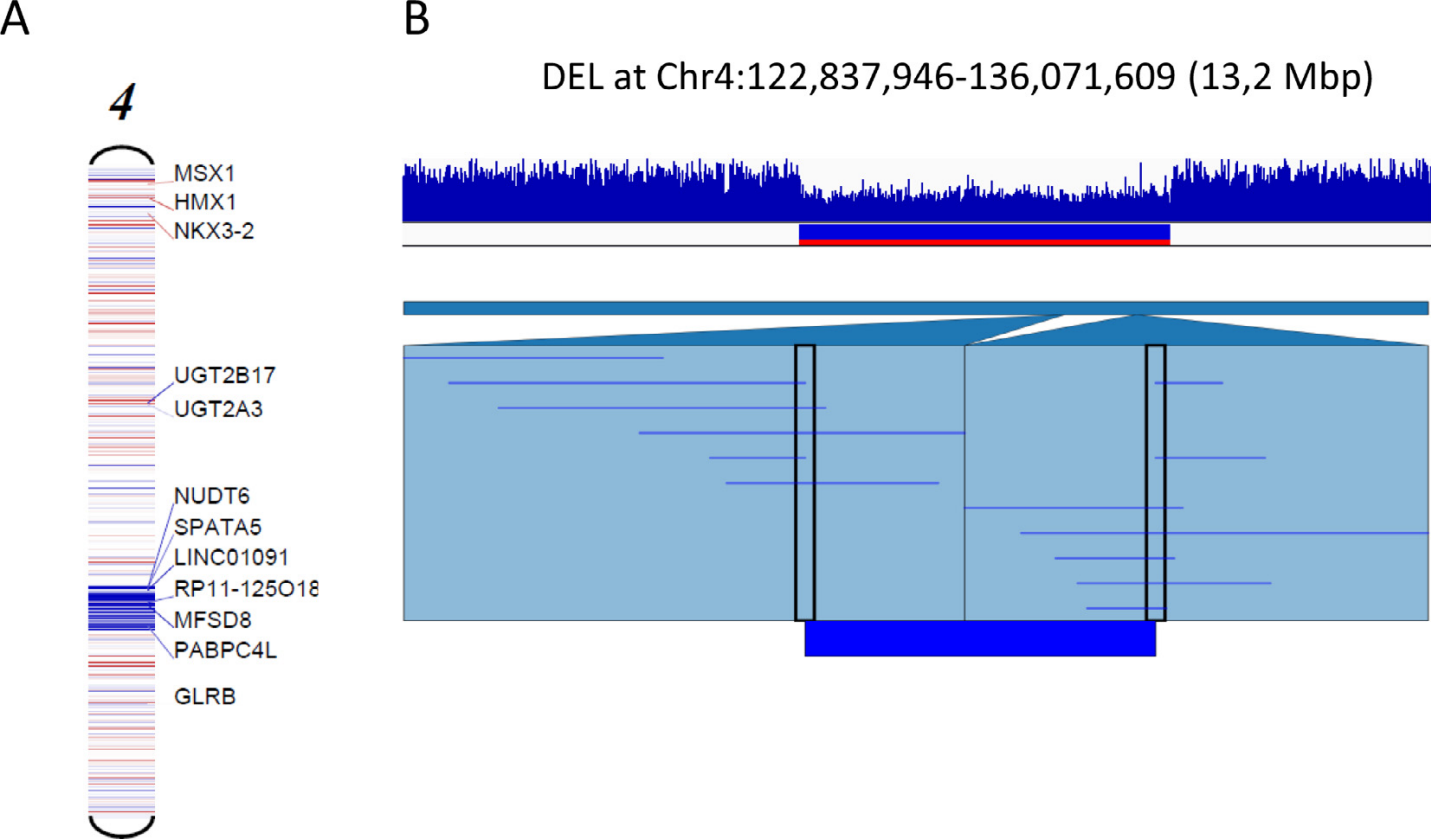
A 13.2 Mb large deletion on chromosome 4. (A) Detection and identification by CNV analysis of the target clinical exome short read sequencing data. (B) Identification by long reads sequencing.

Simple large chromosomal rearrangements, like multi-locus deletions, are easy to be determined. Fig. 1 illustrates an 13.2 Mb deletion that was successfully detected and identified using either short or long read data (Fig. 1). In any case, following the SV detection of interest, visualization should be optimal and plays a pivotal role to determine which genes (or exons) are involved in the structural rearrangement. Integrative Genomics Viewer (IGV) is a commonly used tool for the interactive exploration of reference-based aligned data and SVs [25]. Furthermore, the Ribbon tool (genomeribon.com) displays the alignments along the reference and query sequences nicely, together with any associated variant calls in the sample [26].

### 3.2 *De novo assembly* followed by reference-based assembly alignment

To complement reference-based alignment with variant calling, *de novo* assembly can also identify the structure of nested SVs. In such case, Canu, wtbg2, and FALCON are frequently used tools for the *de novo* assembly of long reads. Canu and wtdbg2 can assemble long nosy reads produced by ONT and PacBio sequencing, while FALCON can assemble PacBio reads only. Wtbg2 is using the fuzzy Bruijn graph approach when assembling the human genome, which has a great advantage of being tens of times faster than Canu and FALCON, while producing contigs of comparable base accuracy [27]. However, to uncover the diploid nature of the genome and thus, the heterozygous large complex SVs, the user needs to construct a diploid genome assembly. Haploid assemblers mostly collapse the two sequences into one haploid consensus sequence that arbitrarily alternates between both alleles [28]. Consequently, heterozygous variants are misidentified, as they are left out of an assembly or are represented only as alternate contig sequences. FALCON and FALCON-Unzip are used to assemble long PacBio reads into a highly accurate, contiguous, and correctly phased diploid genome assembly. FALCON use reads to construct a string graph that contains sets of “haplotype-fused” contigs as well as bubbles, representing divergent regions between homologous sequences. In addition, FALCON-Unzip forms the final diploid assembly and uses phasing information from heterozygous positions [29]. Furthermore, Canu is a widely used assembler connecting three stages: correction, trimming and assembly. The correction step aligns long reads to each other and thus, selects the best overlaps to use for correction. Then, the trimming stage identifies the unsupported regions in the input and trims and splits reads to their longest supported range. During the assembly stage, Canu makes the final pass to identify sequencing errors and next, constructs the best overlap graph [30]. To construct a diploid genome, Canu has recommendations how to set options when dealing with polyploid genomes, where one option is to avoid collapsing the genome and thus, ending up with double the size of the genome. Canu has also an option to produce the complete assembly of parental haplotypes with trio binning. It uses short reads from two parental genomes to partition long reads from an offspring into haplotype-specific sets prior to the assembly. Each haplotype is then assembled independently, resulting in a complete diploid reconstruction [31].

After having a consensus sequence, the next step is to align it to the reference genome and investigate, if the structure of the rearrangement(s) in question can be assessed. Genome sequence aligner nucmer (part of the MUMmer system) has been widely applied to align whole genome sequences, compare different assemblies of the same genome and align reads to the reference, even though it is less sensitive and accurate than the dedicated read aligners [32]. Additionally, mummerplot with delta-filer enables an informative visualization of the assembly alignment to the reference. With a diploid assembly of good quality, which has large complex SVs included into contigs, the user can precisely solve the length and the structure of the rearrangement in question. The high-resolution visualization of inversions, misassemblies and translocations can also be nicely generated by QUAST. QUAST applies nucmer to align assemblies to a reference genome followed by the quality evaluation of the assemblies by calculating specific metrics, including misassemblies and SVs (to name a few, the number of misassemblies, the assembled contigs length, the number of the unaligned contigs or the number of the ambiguously mapped contigs) [33]. Finally, Assemblytics uses the delta file produced by nucmer to detect and analyse variants from a *de novo* genome assembly aligned to a reference genome. Assemblytics can identify all the insertions and deletions from 1b up to a maximum 10 kb in size. The maximum limit is defined by the minimum amount of the unique contig sequence anchor, contained in no other alignments of that contig. In that way, it prevents translocations and complex variants from being interpreted as indels [34].

## 4 Hybrid approaches to the rescue

When thinking of important technological advances for the discovery and identification of SVs, BioNano optical mapping, 10x Genomics or chromatin conformation capture (Hi-C) crosslinking protocols should not be overlooked (Table 2).

**Table 2:** Long range sequencing and mapping platforms.

Platform	General characteristic	Key features for the determination of SVs	Limitations for the determination of SVs
Long reads sequencing (Oxford Nanopore Sequencing, PacBio SMRT sequencing)	Single-molecule long read sequencing averaging ∼10 k	Single reads spanning whole SV or its break points	Large quantities of high molecular weight DNA High error rate
BioNano Genomics optical mapping	Optical mapping of long DNA reads ∼250 kb or longer	Single molecule spanning structural variants > 10 kb	Does not provide a nucleotide-level resolution of breakpoints
10X Genomic Chromium	Linked short reads spanning ∼100 kb	Linked reads spanning ∼100 kb can detect large SV variants > 10 kb	Unable to identify complex inversions
Hi-C based analysis	Pairs of short reads formed from crosslinking chromatin interactions	Chromatin contact maps determining large SV with reads spanning breakpoints and reads located nearby the breakpoints	Limited in detecting SVs within 1 MB scale Does not provide a nucleotide-level resolution of breakpoints
Strand-Seq	Single-cell/single-strand genome sequencing	Possible to identify, haplotypes and h genomic rearrangements including complex inversions	High cost and demanding procedure (the protocol requires viable mitotic cells)

PacBio SMRT, Pacific Biosciences single-molecule real time.

BioNano Genomics combines long-read technology with low resolution sequencing. Enzymes nick and fluorescently label specific sequences within DNA fragments that are up to ∼1 Mb long. Then, fragments are assembled and/or aligned to the reference genome to map the locations of the probes in question. This approach can identify SVs that span up to tens of kb, however it does not provide a nucleotide-level resolution. For detecting the precise structure of genomic rearrangements, BioNano optical mapping can serve as a good companion to NGS technologies by providing a long-range scaffold to *de novo* genome assemblies [35]. Due to the error prone specifics of long reads, optical mappings are mostly used in combination with either short read data or linked read data [36], [37]. On the other hand, optical mapping combines signals, so that only the summed effect may be measured, when two or more SVs are within a given pair of cleavage sites, making it difficult to assess complex chromosomal rearrangements [15].

A multi-platform comparison between BioNano optical mappings, Illumina short read sequencing and PacBio long read sequencing revealed that insertions and deletions between 10 kb and 1 Mb are most accurately detected by BioNano optical mapping. Insertions between 1 kb and 5 kb can be detected by BioNano, PacBio as well as their synergy, whereas deletions can be identified either with short-reads or long-reads as well as by BioNano optical mapping. Additionally, median size insertions (between 50 b and 1 kb) are mostly detected by PacBio, while some deletions can be detected only with Illumina short reads. Large inversions (>50 kb) were detected only by single-cell/single-strand genome sequencing [38]. The latter can distinguish forward from reverse strands based on their 5′-3′ orientation. For each chromosome within the cell, this method can determine the inheritance patterns for each DNA template strand. An inversion can be observed as homozygous or heterozygous, but the structure of a nested rearrangement cannot be identified.

10x Genomics or Hi-C crosslinking protocols can also determine a *de novo* assembly and hence, SV structures, as they are both coupled with short read sequencing to provide base-pair-resolution. The chromium technology from 10x Genomics enables identification determination of a diploid genome sequence at high resolution. It does so, by partitioning large DNA fragments into micelles, which typically contain < 0.3x copies of the genome and one unique barcode. In each micelle, smaller fragments are amplified and barcoded, afterwards the pooled DNA undergoes a standard library preparation and sequencing. The reads are aligned and linked together to form a series of anchored fragments, which can span up to 100 kb in length [35], [39]. Furthermore, entire eukaryotic chromosomes as well as chromosomal rearrangements were resolved using high-quality draft assembly, produced by short- or long- read sequencing in combination with Hi-C crosslinking protocols. This is a chromosome conformation capture-based technique, which simultaneously captures long-range interactions among pairs of fragments and fragment-specific nucleotide sequence​ [40], [41], [42], [43].

## 5 Functional studies

Experiencing the era of big data and technological advances, a series of wet- and dry-lab approaches hold the promise of translating information growth into knowledge growth. In this context, synergies play a pivotal role, in particular if clinical relevance and cost-effectiveness are considered; multi-omics may map inter-individual variability via holistic profiling, 3D cell (co)cultures may dissect molecular mechanisms and provide mechanistic insight, and information technologies may inform decision-making.

Upon interpretation of complex SV, prominent key questions go beyond inferring their architecture, questioning their role (if any). To this end, reconstructing and visualizing such complex variant structures is not trivial, while functional predictions remain a bottleneck. Looking for sustainable and cost-effective strategies given the scale of current and forthcoming genome sequencing endeavours, one might consider the synergy of artificial and human intelligence [44]. Humans can detect patterns, which computer algorithms may fail to do so, whereas data-intensive and cognitively complex settings and processes limit human ability [45]. We feel that it is highly likely complex SVs are more prevalent, and more architecturally diverse, than currently recognized due to under-ascertainment and misinterpretation. To date, the accuracy of interpretation depends entirely on the accuracy of the underlying breakpoint calls, and hence, current breakpoint mapping strategies suffer from high false negative or positive rates or both [46], [47], [48].

Mechanistically minded studies aim to reconstruct the mutational events that resulted in the SV of interest, as already experienced in ancestral genome reconstruction using breakpoint graphs [49], [50], and for inferring the mutational history of segmental duplications by modified A-Bruijn graphs [51] or DAWGs [52]. Despite genome-scale models are subjected to simplifying assumptions to prevent computational complexity, optimal pipelines should be possible for any given complex variant. How are such optimal strategies defined? Taking into account current mutation models, this answer remains challenging.

Karyotyping with or without FISH are considered effective ways of identifying large scale structural variation, despite their relatively low resolution [53]. Genome wide Hi-C, which was developed to identify spatial genome organization [54], [55] is emerging as a tool for identifying structural variants [40], [41] as well as de-novo genome assembly [56]. Jacobson et al performed Hi-C and RNA-sequencing to identify and compare large SVs in HL-60 and HL-60/S4 cell lines and validated the accuracy of their approach [42]. A framework that integrates optical mapping, Hi-C and whole-genome sequencing was employed to resolve complex SVs and phase multiple SV events to a single haplotype [40]. Notably, noncoding SVs raise concerns as they may be underappreciated mutational drivers in cancer genomes.

Multi-omics could be of great benefit in resolving the enigma of the functional role of SVs. A multi-omics design was employed to explore the presence of SVs in heart failure patients due to dilated cardiomyopathy, in which genomic aberrations were linked to myocardial gene expression by performing heart-specific SV-eQTL and SV-load correlations [57]. In the same study, high-density methylation arrays, PCR-based and nanopore sequencing were coupled to transverse aortic constriction to investigate potential dysregulation of SV-eQTL homologous transcripts in mice with induced heart failure [40]. Zook et al. integrated sequence-resolved SV calls from diverse technologies and SV calling approaches towards a benchmark for germline SV detection enabling the assessment of both false negative and false positive rates. The authors aimed to evaluate SV accuracy from essentially any genomic technology, including short, linked, and long read sequencing technologies, optical mapping and electronic mapping [48].

3D cell co-cultures may address the challenge of heterogeneous cell mixtures with possibly different numbers of mutations. Cancer serves as a paradigm, as admixture between normal and tumor cells is present or cell subpopulations that may contain a range of SVs, including driver or drug resistance mutations. Despite single cell technologies [58], the signal for detecting variants in the majority of current sequencing efforts is proportional to the number of cells in the mixture that contain that variant and therefore, the normal cells present will reduce the power to detect somatic mutations. Furthermore, the detection of rare mutations in the tumor cell population will be even lower [59]. 3D cell co-cultures not only enable in-depth single cell phenotyping, but also allows cell-to-cell mapping minimizing artefacts [60], [61].

## 6 A clinical example

**Fig. 2:**
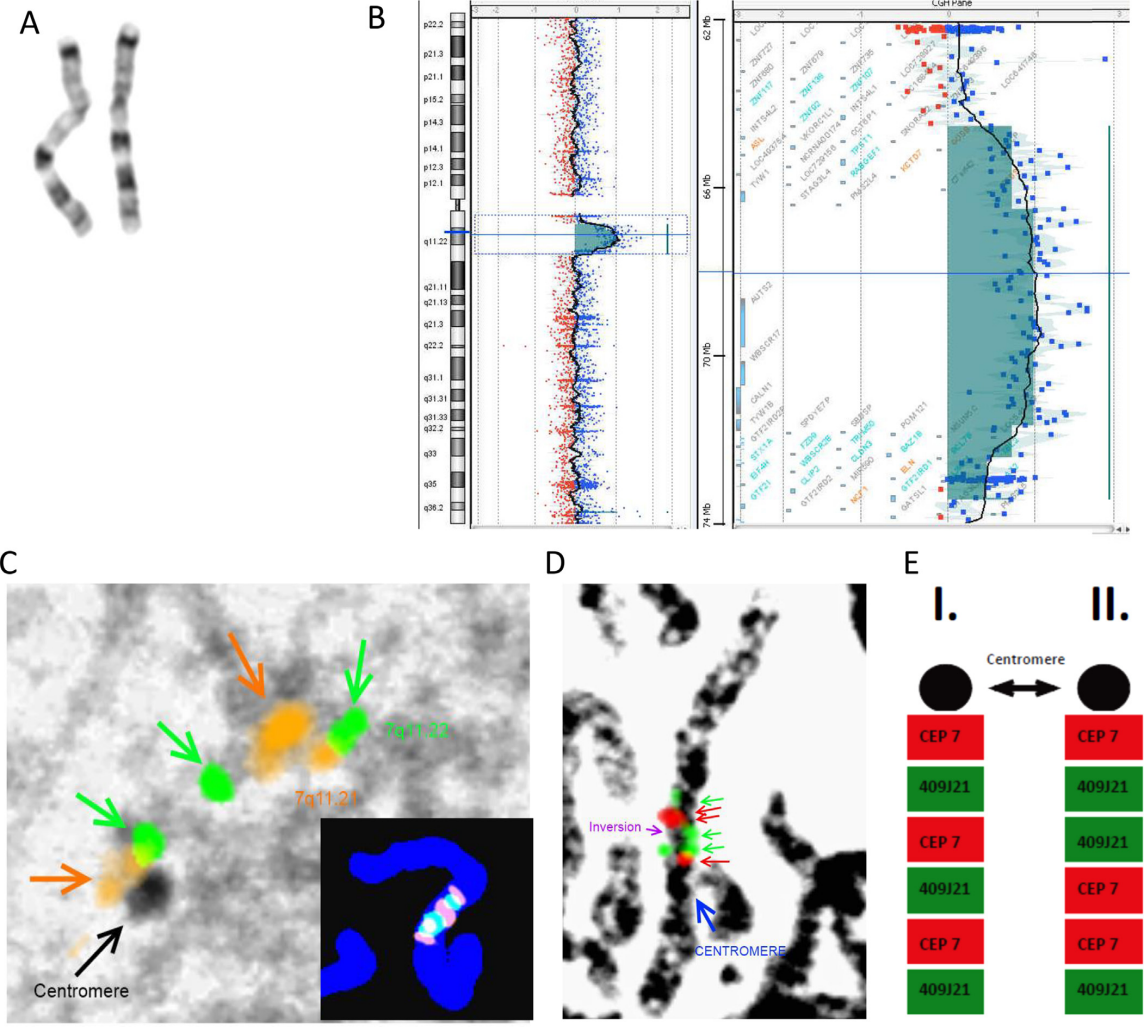
Identification of a large complex SV by a synergy of cytogenetic approaches. (A) The observed G-banded karyotype. (B) The result of aCGH indicating the 1.93 Mb duplication of segment 7q11.21, 5.27 Mp triplication of segment 7q11.21q11.22 and 1.33 Mb duplication of segment 7q11.23. (C) The 7q11.22 triplication is detected by FISH. The regions 7q11.21 (orange), 7q11.22 (green) and 7q11.23 (orange) are shown by combined FISH. (D) The result of FISH inversion detection using distal (RP11-409J21) and proximal (CEP 7, Agilent) probes. (E) Two different possible scenarios: I. if the colour pattern oscillates between red and green, the inversion is not present, II.: the presence of inversion is confirmed. (For interpretation of the references to colour in this figure legend, the reader is referred to the web version of this article.)

Our pipeline was employed to resolve a clinical case where a large structural rearrangement was observed by G-banded karyotype (Fig. 2, A), followed by the identification of a large triplication with duplications upon screening for large insertion(s) or deletion(s) using aCGH (Fig. 2, B). Mapping the chromosomal regions 7q11.21, 7q11.22, and 7q11.23 by multiple combinations of specific FISH probes, the triplication was validated and confirmed (Fig. 2, C), while an extra inversion was detected (Fig. 2, D). Thus, an inverted triplication of 7q11.22 embedded within the 7q11.21q11.23 duplication segment was proposed.

Taking into account that the analysis of large complex rearrangements and high-resolution breakpoints profiling remain difficult, cytogenetic approaches do not suffice and hence, multi-step synergies of state-of-the-art genomic sequencing and mapping technologies are emerging to shed light on clinical phenotypes.

**Fig. 3:**
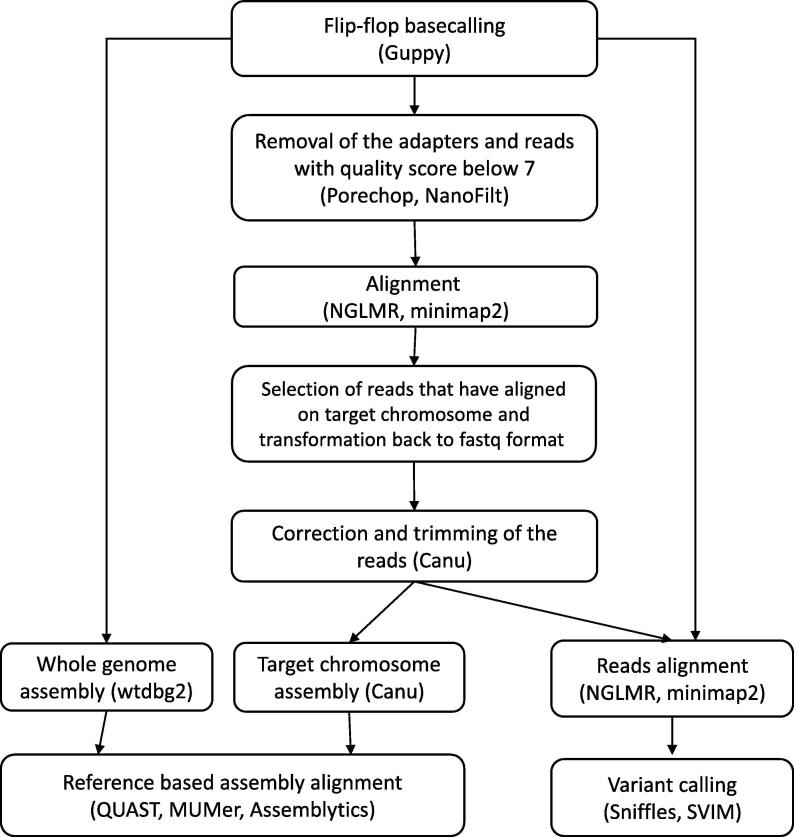
The bioinformatics pipeline set herein for the analysis of the large complex SV in question by Nanopore MinION technology. Best identification results were acquired via the synergy of reference alignment and *de novo* assembly approaches.

Nanopore MinION technology was applied to determine the precise variant configuration of the large complex SV previously observed by G-banded karyotype, aCGH and FISH. Median read quality was 12.44, representing a 13.2x theoretical coverage of the human genome, with an average N50 read length of 10.2 kb. Currently, there is no gold standard bioinformatics approach for detecting and identifying SVs with long reads, especially when the chromosomal rearrangement in question is few Mbp in length. To identify the structural variant(s) and break points that could explain the underlying chromosomal rearrangement in the clinical case in question several computational approaches have been explored (Fig. 3).

**Fig. 4:**
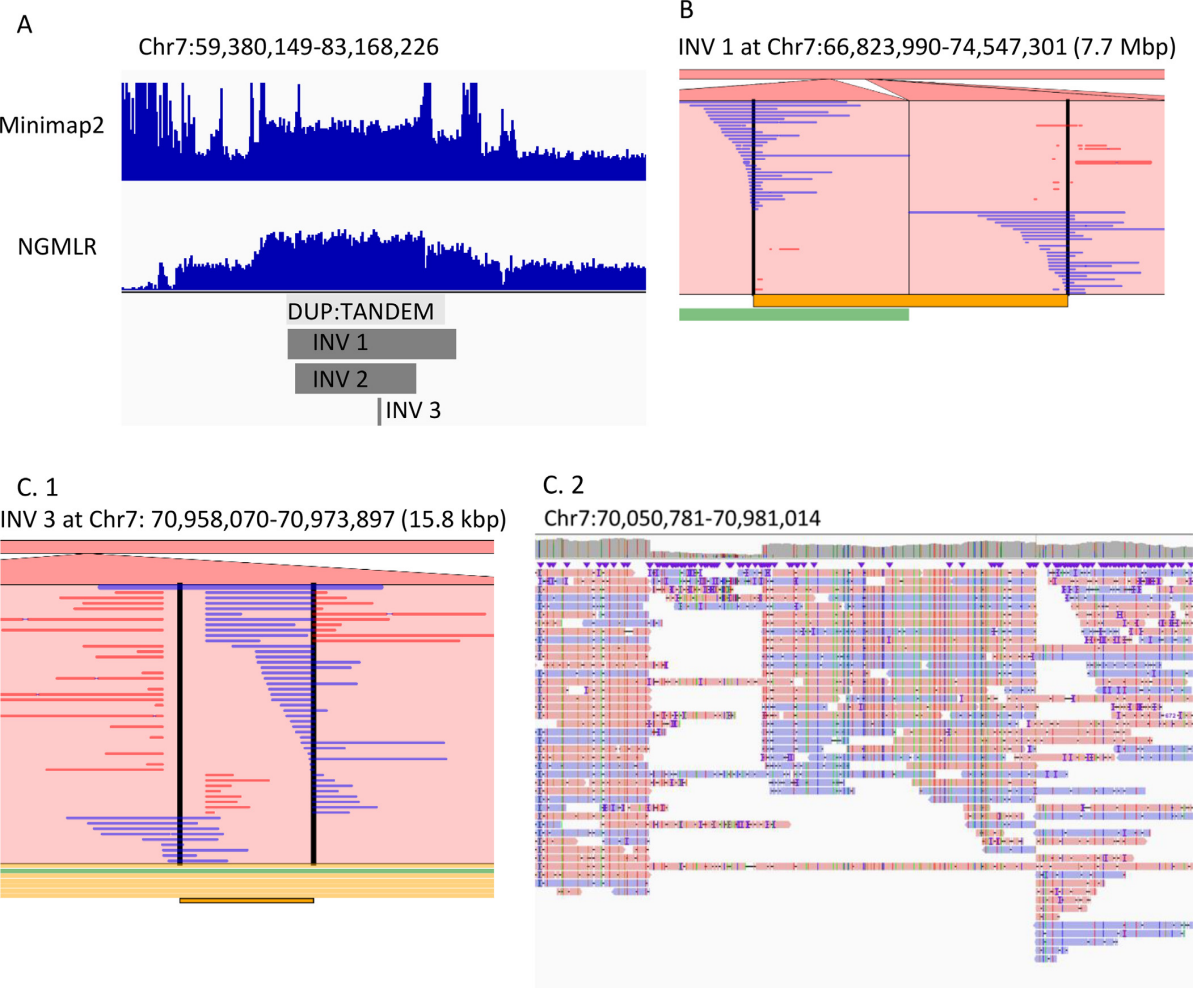
Visualization of the large complex SV in question, following the application of long reads sequencing. (A) The gain in the reads coverage of 7q11.21q11.23q21.11 obtained by Minimap2 aligner and NGMLR mapper and the detected SVs. (B) The Ribbon view of the reads spanning the 7.7 Mb large inversion. (C.1) The Ribbon and (C.2) IGV views of the reads spanning the 15.8 kb large inversion.

A read depth analysis was performed (Fig. 4, A) to define and confirm with high resolution a gain in the reads coverage observed with aCGH (Fig. 1, B). Read-depth analysis can identify the gain for triplication and duplication, yet the precise breakpoints cannot be defined (reads coverage variation). Our findings on read coverage were inconsistent when probable breakpoints were explored by the NGMLR mapper and Minimap2 aligner (Fig. 4 A); a gain in read coverage was obtained by Minimap2 vs. NGMLR, which revealed a lower number of reads on these areas. Such discrepancies may be attributed to the specifics of each algorithm for reads splitting at breakpoints. Upon aligning the reads to the reference genome by the NGMLR mapper or Minimap2 aligner and next, variant calling by Sniffles or SVIM (with parameter optimization for a minimum SV size of 1000 and maximum SV size of 10,000,000), we did not detect any variants that could explain the observed read coverage gain in question. However, variant calling with SVIM on the reads aligned with the NGMLR mapper revealed one inversion (namely, INV 2 on Fig. 4, A). Moreover, to overcome the high frequency of errors in long reads, we have used Canu self-correction and a trimming step. Since it is estimated that Canu needs 20,000 CPU hours to assemble the whole human genome, we have selected only reads that have aligned on chromosome 7. Because of the different alignment of reads on probable breakpoints, a slightly different set of reads was selected from the NGMLR mapper or Minimap2 aligner. After NGLMR alignment, we have detected a few probable inversions and one tandem duplication by SVIM (Fig. 4) and a probable duplication by Sniffles in the area of interest. Neither of the variant callers used on the reads aligned by Minimap2 resulted in any large SV that could explain the rearrangement under investigation, thereupon a higher coverage would be needed. Overall, applying the reference-based alignment approach, we empowered long reads technology and demonstrated the detection of a few kb large inversions, insertions or deletions. When many reads span along the whole SV, the breakpoint can be clearly seen with base-pair-resolution in IGV (Fig. 4, C.2) or Ribbon (Fig. 4, C.1). However, when a structural variation is nested and much larger than the average read, it is still a great challenge to resolve the complex SV structure and determine the precise breakpoints (Fig. 4, B).

In addition to true SVs, we have also observed many large false positives SVs detected by any combination of aligner and variant caller. To our knowledge, CNV detection for long read whole genome sequencing is not yet available, thus pointing towards the need to combine long read sequencing with cytogenetic or optical mapping approaches to better define the structural rearrangement(s) and region of interest. Assembly approaches did not give us any additional valuable information, most probably because of insufficient coverage. In diagnostics, reaching a high coverage as 50x or more with Nanopore technology is still costly and requires a relatively higher amount (up to 10^3) of high molecular weight DNA samples in comparison to short read sequencing. As shown in our case study, it remains difficult to ensure a sufficient amount of DNA to acquire optimal coverage. No doubt, continuous optimization of the library preparation protocols as well as sequencing pipelines are in place, with the aim to lower required DNA input for the same data quality.

## 7 Summary and outlook

The success in the identification of genomic structural rearrangement(s) in routine clinical protocols mainly depends on the complexity and size of SVs. Short and/or simple SV are being successfully identified by cytogenetic techniques or short read sequencing, while large nested and complex rearrangements demand case-specific investigation via the application of novel emerging technologies as those presented in our clinical example. A clinical phenotype of unexplained severe DD or DD with multiple embedded or associated gain/loss genomic events identified by aCGH may be indicative for long-read sequencing application, accompanied by the presented bioinformatics approaches. The identification of the exact composition of the underlying structural rearrangement may improve treatment and prognosis counselling as well as potential future family planning. Such novel technologies will be of great benefit when standardized and validated analytical protocols become widely available. There is still a missing gap in guidelines and standards for identifying the detailed composition of large structural rearrangements. When facing a rare few Mbp in size nested SV it is difficult to decide which approach to use to provide the most suitable diagnosis to the patient. Long read sequencing carries a huge potential to become the routinely used technology for identifying large structural rearrangements in clinical diagnostics, yet several challenges need to be resolved, among others, increasing the average length of the reads ideally encompassing the whole region of the rearrangements of interest. Of note, the technology should be cost-effective, to be of benefit to a health care. To set a benchmark system, herein, we performed cytogenetic screening with low resolution, first, to select cases where long read sequencing would be of benefit. Notwithstanding, relatively high error rates are still a bottleneck in genetic testing by long reads.

## Declaration of Competing Interest

The authors declare that they have no known competing financial interests or personal relationships that could have appeared to influence the work reported in this paper.

## References

[1] Sudmant P.H., Rausch T., Gardner E.J., Handsaker R.E., Abyzov A., Huddleston J. (2015). An integrated map of structural variation in 2,504 human genomes. Nature.

[2] Tjio J.H. (1978). The chromosome number of man. Am J Obstetrics Gynecol.

[3] Kannan T.P., Zilfalil B.A. (2009). Cytogenetics: past, present and future. Malaysian J Med Sci: MJMS.

[4] Drets M.E., Shaw M.W. (1971). Specific banding patterns of human chromosomes. PNAS.

[5] Landegent JE, Jansen in de Wal N, van Omment G-JB, Baas F, de Vijlderi JJM, van Duijn P, et al. Chromosomal localization of a unique gene by non-autoradiographic in situ hybridization. Nature 1985;317(6033):175–177. doi: 10.1038/317175a0. 10.1038/317175a0 3839907

[6] Riegel M. (2014). Human molecular cytogenetics: from cells to nucleotides. Genet Mol Biol.

[7] Arppe R., Carro-Temboury M.R., Hempel C., Vosch T., Just Sørensen T. (2017). Investigating dye performance and crosstalk in fluorescence enabled bioimaging using a model system. PloS One.

[8] Balajee A.S., Hande M.P. (2018). History and evolution of cytogenetic techniques: Current and future applications in basic and clinical research. *Mutat Res Genet Toxicol Environ Mutagen*.

[9] Kallioniemi A, Kallioniemi OP, Sudar D, Rutovitz D, Gray JW, Waldman F, et al. Comparative genomic hybridization for molecular cytogenetic analysis of solid tumors. Science 1992;258(5083):818 LP–821. doi: 10.1126/science.1359641. 10.1126/science.1359641 1359641

[10] Wicker N., Carles A., Mills I.G., Wolf M., Veerakumarasivam A., Edgren H. (2007). A new look towards BAC-based array CGH through a comprehensive comparison with oligo-based array CGH. BMC Genomics.

[11] Ramos L., del Rey J., Daina G., García-Aragonés M., Armengol L., Fernandez-Encinas A. (2014). Oligonucleotide arrays vs. metaphase-comparative genomic hybridisation and BAC arrays for single-cell analysis: first applications to preimplantation genetic diagnosis for Robertsonian translocation carriers. PloS One.

[12] Coughlin C.R., Scharer G.H., Shaikh T.H. (2012). Clinical impact of copy number variation analysis using high-resolution microarray technologies: advantages, limitations and concerns. Genome Med.

[13] Jain M., Olsen H.E., Paten B., Akeson M. (2016). The Oxford Nanopore MinION: delivery of nanopore sequencing to the genomics community. Genome Biol.

[14] Roberts R.J., Carneiro M.O., Schatz M.C. (2013). The advantages of SMRT sequencing. Genome Biol.

[15] Sedlazeck F.J., Lee H., Darby C.A., Schatz M.C. (2018). Piercing the dark matter: bioinformatics of long-range sequencing and mapping. Nat Rev Genet.

[16] Rang F.J., Kloosterman W.P., de Ridder J. (2018). From squiggle to basepair: computational approaches for improving nanopore sequencing read accuracy. Genome Biol.

[17] Wick R.R., Judd L.M., Holt K.E. (2019). Performance of neural network basecalling tools for Oxford Nanopore sequencing. BioRxiv.

[18] Weirather JL, de Cesare M, Wang Y, Piazza P, Sebastiano V, Wang X-J, et al. Comprehensive comparison of Pacific Biosciences and Oxford Nanopore Technologies and their applications to transcriptome analysis. F1000Research 2017;6:100. doi: 10.12688/f1000research.10571.2. 10.12688/f1000research.10571.1 PMC5553090 28868132

[19] De Coster W., De Rijk P., De Roeck A., De Pooter T., D’Hert S., Strazisar M. (2019). Structural variants identified by Oxford Nanopore PromethION sequencing of the human genome. Genome Res.

[20] Li H. (2018). Minimap2: pairwise alignment for nucleotide sequences. Bioinformatics (Oxford, England).

[21] Sedlazeck F.J., Rescheneder P., Smolka M., Fang H., Nattestad M., von Haeseler A. (2018). Accurate detection of complex structural variations using single-molecule sequencing. Nat Methods.

[22] Heller D., Vingron M. (2019). SVIM: structural variant identification using mapped long reads. Bioinformatics.

[23] English A.C., Salerno W.J., Reid J.G. (2014). PBHoney: identifying genomic variants via long-read discordance and interrupted mapping. BMC Bioinf.

[24] Huddleston J., Chaisson M.J.P., Steinberg K.M., Warren W., Hoekzema K., Gordon D. (2017). Discovery and genotyping of structural variation from long-read haploid genome sequence data. Genome Res.

[25] Robinson J.T., Thorvaldsdóttir H., Winckler W., Guttman M., Lander E.S., Getz G. (2011). Integrative genomics viewer. Nat Biotechnol.

[26] Nattestad M., Chin C.-S., Schatz M.C. (2016). Ribbon: visualizing complex genome alignments and structural variation. BioRxiv.

[27] Ruan J., Li H. (2019). Fast and accurate long-read assembly with wtdbg2. BioRxiv.

[28] Church D.M., Schneider V.A., Steinberg K.M., Schatz M.C., Quinlan A.R., Chin C.-S. (2015). Extending reference assembly models. Genome Biol.

[29] Chin C.-S., Peluso P., Sedlazeck F.J., Nattestad M., Concepcion G.T., Clum A. (2016). Phased diploid genome assembly with single-molecule real-time sequencing. Nat Methods.

[30] Koren S., Walenz B.P., Berlin K., Miller J.R., Bergman N.H., Phillippy A.M. (2017). Canu: scalable and accurate long-read assembly via adaptive k-mer weighting and repeat separation. Genome Res.

[31] Koren S., Rhie A., Walenz B.P., Dilthey A.T., Bickhart D.M., Kingan S.B. (2018). Complete assembly of parental haplotypes with trio binning. BioRxiv.

[32] Marcais G., Delcher A.L., Phillippy A.M., Coston R., Salzberg S.L., Zimin A. (2018). MUMmer4: a fast and versatile genome alignment system. PLoS Comput Biol.

[33] Gurevich A., Saveliev V., Vyahhi N., Tesler G. (2013). QUAST: quality assessment tool for genome assemblies. Bioinformatics (Oxford, England).

[34] Nattestad M., Schatz M.C. (2016). Assemblytics: a web analytics tool for the detection of variants from an assembly. Bioinformatics.

[35] Goodwin S., McPherson J.D., McCombie W.R. (2016). Coming of age: ten years of next-generation sequencing technologies. Nat Rev Genet.

[36] Eisfeldt J., Pettersson M., Vezzi F., Wincent J., Käller M., Gruselius J. (2019). Comprehensive structural variation genome map of individuals carrying complex chromosomal rearrangements. PLoS Genetics.

[37] Mostovoy Y., Levy-Sakin M., Lam J., Lam E.T., Hastie A.R., Marks P. (2016). A hybrid approach for de novo human genome sequence assembly and phasing. Nat Methods.

[38] Chaisson M.J.P., Sanders A.D., Zhao X., Malhotra A., Porubsky D., Rausch T. (2019). Multi-platform discovery of haplotype-resolved structural variation in human genomes. Nat Commun.

[39] Weisenfeld N.I., Kumar V., Shah P., Church D.M., Jaffe D.B. (2017). Direct determination of diploid genome sequences. Genome Res.

[40] Dixon J.R., Xu J., Dileep V., Zhan Y., Song F., Le V.T. (2018). Integrative detection and analysis of structural variation in cancer genomes. Nat Genet.

[41] Harewood L., Kishore K., Eldridge M.D., Wingett S., Pearson D., Schoenfelder S. (2017). Hi-C as a tool for precise detection and characterisation of chromosomal rearrangements and copy number variation in human tumours. Genome Biol.

[42] Jacobson E.C., Grand R.S., Perry J.K., Vickers M.H., Olins A.L., Olins D.E. (2019). Hi-C detects novel structural variants in HL-60 and HL-60/S4 cell lines. Genomics.

[43] Redin C., Brand H., Collins R.L., Kammin T., Mitchell E., Hodge J.C. (2017). The genomic landscape of balanced cytogenetic abnormalities associated with human congenital anomalies. Nat Genet.

[44] Katsila T., Konstantinou E., Lavda I., Malakis H., Papantoni I., Skondra L. (2016). Pharmacometabolomics-aided pharmacogenomics in autoimmune disease. *EBioMedicine*.

[45] Agrawal D, Bernstein P, Bertino E, Davidson S, Dayal U, Franklin M, et al. Challenges and Opportunities with Big Data – A community white paper developed by leading researchers across the United States. March 2012. Retrieved from http://cra.org/ccc/docs/init/bigdatawhitepaper.pdf.

[46] Mantere T., Kersten S., Hoischen A. (2019). Long-read sequencing emerging in medical genetics. Front Genet.

[47] Quinlan A.R., Hall I.M. (2012). Characterizing complex structural variation in germline and somatic genomes. Trends Genet: TIG.

[48] Zook J.M., Hansen N.F., Olson N.D., Chapman L.M., Mullikin J.C., Xiao C. (2019). A robust benchmark for germline structural variant detection. BioRxiv.

[49] Murphy W.J., Larkin D.M., Everts-van der Wind A., Bourque G., Tesler G., Auvil L. (2005). Dynamics of mammalian chromosome evolution inferred from multispecies comparative maps. Science (New York, N.Y.).

[50] Pevzner P., Tesler G. (2003). Human and mouse genomic sequences reveal extensive breakpoint reuse in mammalian evolution. PNAS.

[51] Jiang Z., Tang H., Ventura M., Cardone M.F., Marques-Bonet T., She X. (2007). Ancestral reconstruction of segmental duplications reveals punctuated cores of human genome evolution. Nat Genet.

[52] Kahn CL, Raphael BJ. A parsimony approach to analysis of human segmental duplications. Pacific Symposium on Biocomputing. Pacific Symposium on Biocomputing 2009:126–137. 19213134

[53] Hasty P., Montagna C. (2014). Chromosomal rearrangements in cancer: detection and potential causal mechanisms. Mol Cell Oncol.

[54] Lieberman-Aiden E., van Berkum N.L., Williams L., Imakaev M., Ragoczy T., Telling A. (2009). Comprehensive mapping of long-range interactions reveals folding principles of the human genome. Science (New York, N.Y.).

[55] Rao S.S.P., Huntley M.H., Durand N.C., Stamenova E.K., Bochkov I.D., Robinson J.T. (2014). A 3D map of the human genome at kilobase resolution reveals principles of chromatin looping. Cell.

[56] Dudchenko O., Batra S.S., Omer A.D., Nyquist S.K., Hoeger M., Durand N.C. (2017). De novo assembly of the Aedes aegypti genome using Hi-C yields chromosome-length scaffolds. Science (New York, N.Y.).

[57] Haas J., Mester S., Lai A., Frese K.S., Sedaghat-Hamedani F., Kayvanpour E. (2018). Genomic structural variations lead to dysregulation of important coding and non-coding RNA species in dilated cardiomyopathy. EMBO Mol Med.

[58] Navin N., Kendall J., Troge J., Andrews P., Rodgers L., McIndoo J. (2011). Tumour evolution inferred by single-cell sequencing. Nature.

[59] Raphael BJ. Chapter 6: structural variation and medical genomics. PLoS Comput Biol 2012;8(12):e1002821. doi: 10.1371/journal.pcbi.1002821. 10.1371/journal.pcbi.1002821 PMC3531322 23300412

[60] Hirschhaeuser F., Menne H., Dittfeld C., West J., Mueller-Klieser W., Kunz-Schughart L.A. (2010). Multicellular tumor spheroids: an underestimated tool is catching up again. J Biotechnol.

[61] Ledur P.F., Onzi G.R., Zong H., Lenz G. (2017). Culture conditions defining glioblastoma cells behavior: what is the impact for novel discoveries?. Oncotarget.

